# Insights in the host response towards biomaterial-based scaffolds for cancer therapy

**DOI:** 10.3389/fbioe.2023.1149943

**Published:** 2023-06-05

**Authors:** Marjolein Schluck, Jorieke Weiden, Martijn Verdoes, Carl G. Figdor

**Affiliations:** ^1^ Department of Tumor Immunology, Radboud Institute for Molecular Life Sciences, Nijmegen, Netherlands; ^2^ Oncode Institute, Nijmegen, Netherlands; ^3^ Institute for Chemical Immunology, Nijmegen, Netherlands

**Keywords:** foreign body response (FBR), synthetic immune niche, cancer immuno therapy, adoptive cell theraphy, cancer vaccine

## Abstract

Immunotherapeutic strategies have shown promising results in the treatment of cancer. However, not all patients respond, and treatments can have severe side-effects. Adoptive cell therapy (ACT) has shown remarkable therapeutic efficacy across different leukaemia and lymphoma types. But the treatment of solid tumours remains a challenge due to limited persistence and tumour infiltration. We believe that biomaterial-based scaffolds are promising new tools and may address several of the challenges associated with cancer vaccination and ACT. In particular, biomaterial-based scaffold implants allow for controlled delivery of activating signals and/or functional T cells at specific sites. One of the main challenges for their application forms the host response against these scaffolds, which includes unwanted myeloid cell infiltration and the formation of a fibrotic capsule around the scaffold, thereby limiting cell traffic. In this review we provide an overview of several of the biomaterial-based scaffolds designed for cancer therapy to date. We will discuss the host responses observed and we will highlight design parameters that influence this response and their potential impact on therapeutic outcome.

## Introduction

Immunotherapy has dramatically changed the treatment of cancer by boosting and steering the anti-tumour immune response (Weber et al., 2020). One important immunotherapeutic strategy is cancer vaccination, which exploits antigen-presenting dendritic cells (DCs) with the aim to enhance the anti-tumour T cell response (Weiden et al., 2018; Weiden et al., 2021). The vaccines provide the DCs with tumour antigens and adjuvants to promote DC activation either by using *ex vivo* DC cultures or by systemic delivery of the activating signals (Sinha et al., 2019), which results in presentation of tumour antigens by DCs and stimulation of tumour-specific T cells. Cancer vaccines have however only provided minimal survival benefits (Lin et al., 2022), which is related to poor persistence of *ex vivo* cultured DCs *in vivo*, inadequate uptake of soluble signals by the DCs *in vivo* and toxicities related to systemic delivery (Sinha et al., 2019). Another promising immunotherapy recently approved by the US Food and Drug Administration is adoptive cell therapy (ACT) of tumour-infiltrating lymphocytes (TILs) or genetically engineered chimeric antigen receptor (CAR-) T cells (Labanieh et al., 2018). CAR-T cell therapy has shown remarkable therapeutic efficacy in the treatment of various B cell malignancies (Maude et al., 2014; Kochenderfer et al., 2015; Lee et al., 2015; Turtle et al., 2016; Zhao and Cao, 2019), but the treatment of solid tumours remains complicated (Sridhar and Petrocca, 2017). (CAR-)T cells are mainly delivered *via* intravenous administration, which complicates the treatment of solid tumours as the (CAR-)T cells encounter challenges in locating and infiltrating these tumours. To improve poor T cell persistence *in vivo*, patients are injected with high doses of cytokine IL-2, which by itself can induce systemic toxicities (Rosenberg, 2014; Weber et al., 2015; Jiang et al., 2016). Moreover, the immunosuppressive tumour microenvironment constrains local (CAR-)T cell expansion (Labanieh et al., 2018). To aid CAR-T cells in finding and infiltrating solid tumours and limit systemic toxicity, local delivery of CAR-T cells directly to the tumour tissue has been investigated (Sridhar and Petrocca, 2017) but a single bolus injection was not found to robustly support CAR-T cell persistence (Sridhar and Petrocca, 2017; Wang et al., 2020). This highlights the need for a delivery vehicle that ensures prolonged persistence of functional (CAR-) T cells to induce a proper anti-tumour immune response.

**FIGURE 1:**
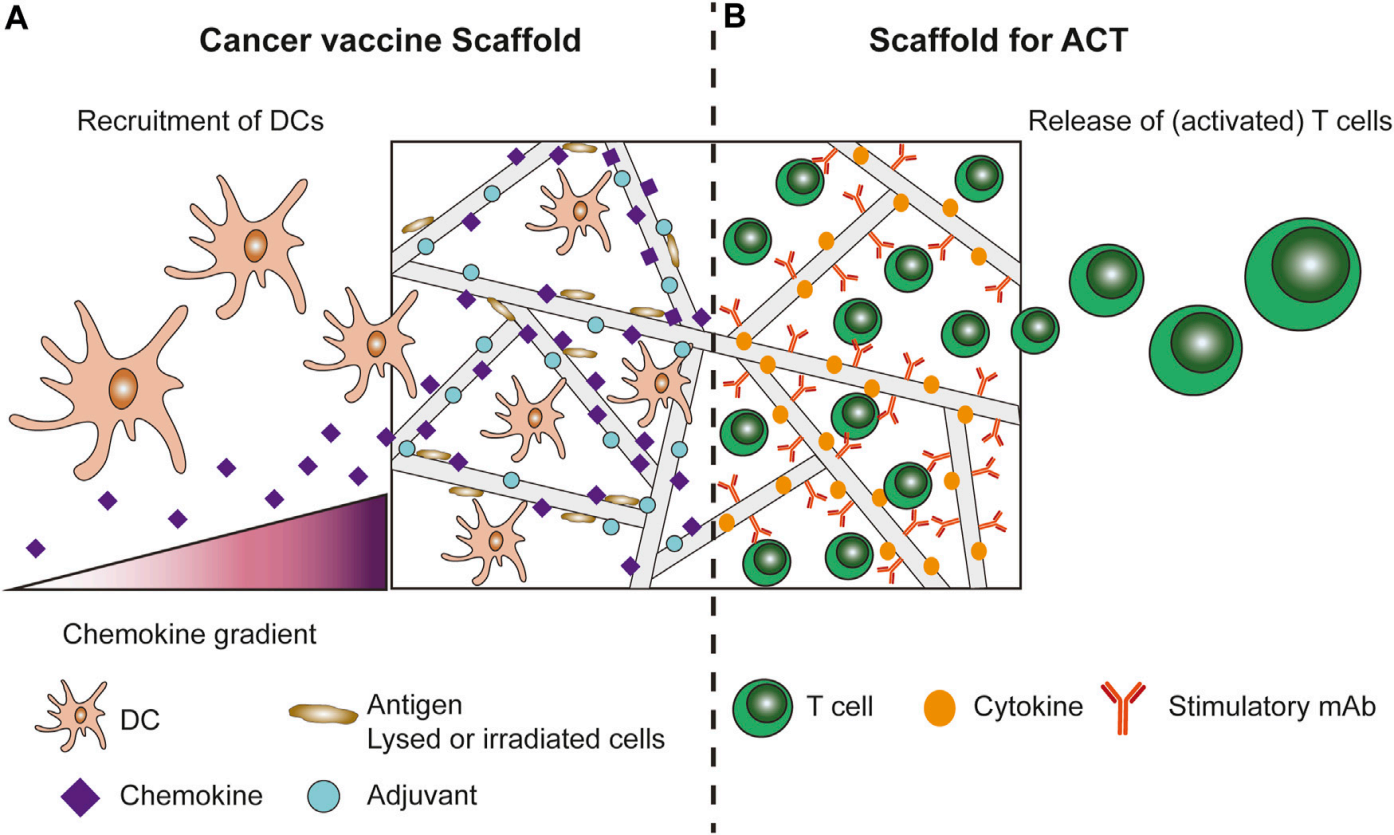
Cancer vaccine scaffolds and scaffolds for ACT of T cells. **(A)** Cancer vaccine scaffolds aim to recruit immune cells, mainly DCs, using a chemokine gradient. Inside the scaffolds the immune cells are provided with a stimulatory 3D environment by incorporating antigens and adjuvants into the scaffold design. **(B)** Scaffolds for ACT of (CAR-)T cells protect the T cells during injection/implantation and provide the T cells with a stimulatory 3D environment *in vivo* to promote better persistence and activation/expansion of the T cells. To this end the scaffolds are modified with stimulatory signals for the T cells, such as cytokines and antibodies.

In recent years, biomaterial-based scaffolds have been designed for their use in tissue engineering and cell delivery. Additionally, scaffolds have been investigated for their role as synthetic immune niches for cancer immunotherapy (Weiden et al., 2018). These scaffolds constitute a 3D environment to locally control the anti-tumour immune response. Synthetic immune niches can be designed as 1) scaffold-based cancer vaccines or 2) to support adoptively transferred T cells (Figure 1). Scaffold-based cancer vaccines create a local immune niche where multiple immunomodulatory signals are provided for prolonged periods of time (Weiden et al., 2018). Moreover, the addition of chemo-attractants to the scaffold enables the recruitment of immune cells, mainly DCs, to the scaffold *in vivo* (Ali et al., 2009; Bencherif et al., 2015; Kim et al., 2015; Verbeke and Mooney, 2015; Verbeke et al., 2017; Shih et al., 2018). Inside the scaffold, DCs are provided with stimulatory signals, such as tumour antigens and adjuvants (Kim et al., 2015; Verbeke et al., 2017). These matured DCs are capable of presenting antigens to T cells to induce T cell activation, either by migrating out of the scaffold towards draining lymph nodes or by interacting with incoming T cells in the scaffold (Weiden et al., 2018). In addition, biomaterial-based scaffolds can be designed to aid local delivery of adoptively transferred (CAR-)T cells (Stephan et al., 2015; Smith et al., 2017; Coon et al., 2020; Wang et al., 2020; Hu et al., 2021; Agarwalla et al., 2022; Grosskopf et al., 2022). These ACT scaffolds create a stimulatory 3D environment for continuous CAR-T cell expansion and activation. One of the advantages of such biomaterial-based scaffolds, compared to systemic approaches, is that the scaffolds can be introduced locally at an intended target site to provide controlled delivery of immunomodulatory agents and/or CAR-T cells. This way, the therapeutic effect can be maximized while the systemic exposure to immunomodulatory agents remains limited (Adu-Berchie and Mooney, 2020). Furthermore, synthetic immune niches can provide the DCs and/or the T cells with the stimulatory signals for prolonged periods of time, and potentially provide immune stimulating signals at much higher doses locally, compared to systemic delivery, while minimizing toxic side effects.

In this review we discuss how the design of these synthetic immune niches can impact the host response against the scaffold. As scaffold-based cancer vaccines are designed to recruit immune cells through incorporation of specific chemotactic factors, which thereby impacts the cellular infiltrate, we will discuss these scaffolds separately from scaffolds designed for ACT. For both scaffold types we will elaborate on their design parameters, host response, and their ability to induce an anti-tumour immune response. We furthermore reflect on findings from the field of tissue engineering (TE) where we focus on the effect that surface modifications can have on the biocompatibility, discussing common findings with scaffolds used for various applications, including TE, cell delivery and, synthetic immune niches. Finally, we picture future directions for optimal scaffold design in local cancer immunotherapy.

### Important scaffold design parameters and host responses

**FIGURE 2:**
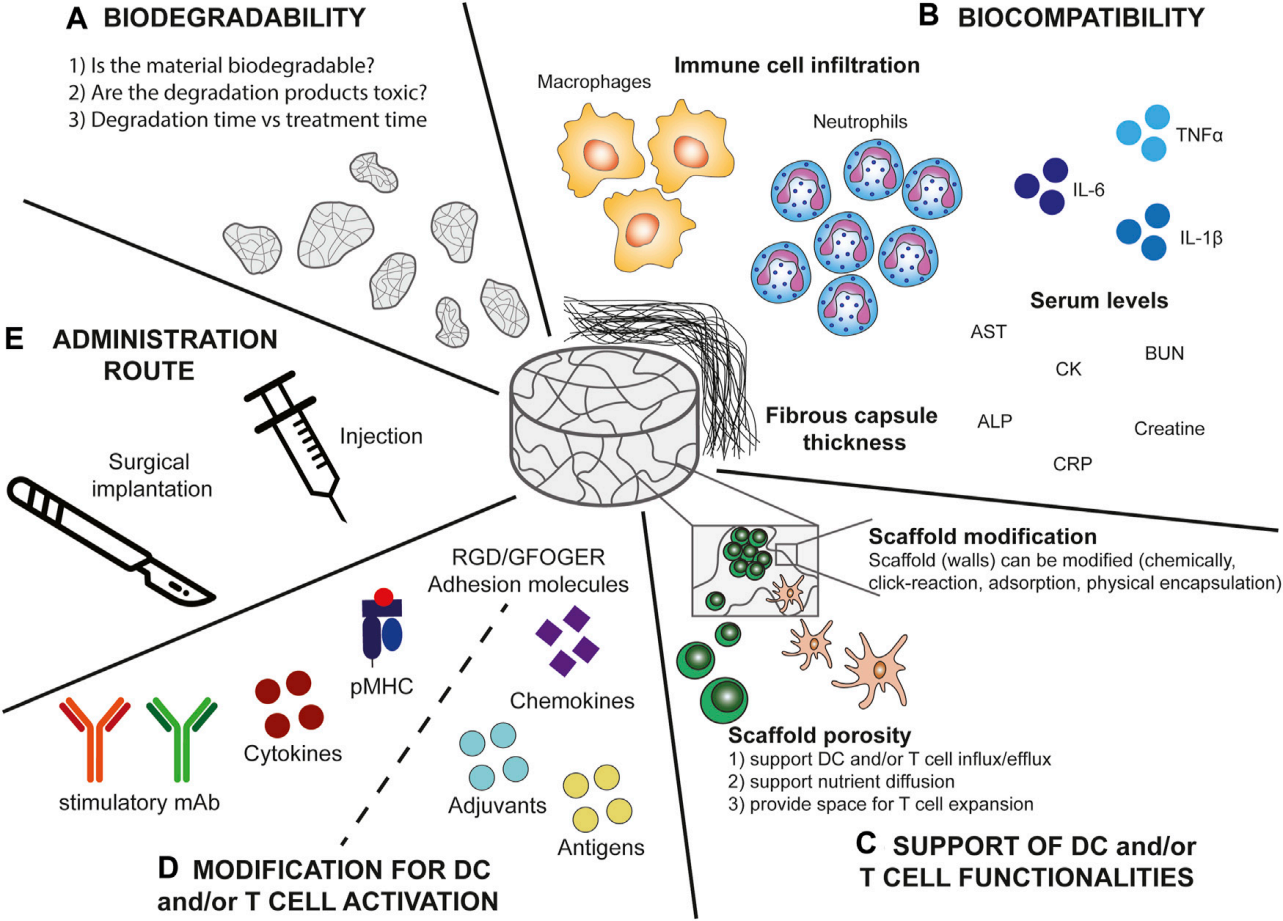
Scaffold parameters important for the design of DC and T cell stimulatory immune niches. **(A)** Biodegradability. When designing biomaterial based-scaffolds it is important to consider whether the material is biodegradable. If the material is not biodegradable this implies that it would need to be (surgically) removed after the treatment. Moreover, when the material is biodegradable, it is important to consider whether the degradation products are toxic or if they can be (easily) cleared by the host cells. Additionally, the degradation time should be in line with the treatment time to prevent unwanted side-effects or treatment failure. **(B)** Biocompatibility. Several factors influence the biocompatibility of the scaffold and the elicited foreign body response of which some are measurable including immune cell infiltration, in particular macrophage and neutrophil infiltration, serum levels of a variety of inflammatory molecules (aspartate aminotransferase (AST), blood urea nitrogen (BUN), alkaline phosphatase (ALP), creatine kinase (CK), and C reactive protein (CRP)), and fibrous capsule thickness. **(C)** Support of DC and/or T cell functionalities. The scaffold should have a good porosity to support DC and/or T cell influx and efflux, cell expansion, persistence, and viability. Additionally, interconnected pores ensure nutrient diffusion into the scaffold. Moreover, the scaffold (walls) should allow for modifications to further support the DCs and/or T cells. **(D)** Modifications of the scaffold for DC and/or T cell activation. The scaffold should provide the opportunity to be modified to provide DCs and/or T cells with stimulatory signals. These include for DCs chemokines for specific recruitment and adjuvants and antigens for DC activation. For T cells these signals include agonistic antibodies, peptide-MHC complexes, adhesion molecules, and cytokines to support T cell functionalities (including cytokine production and tumour cell lysis). **(E)** Administration route. Preferably the scaffold can be delivered by minimally invasive injection to prevent risks accompanied by surgical implantation, however, surgery might provide more controlled placement of the scaffold.

Several parameters are important when considering the design of synthetic immune niches, such as the host response evoked by the scaffold (which is related to its biocompatibility), administration route, biodegradability, mechanical integrity, as well as porosity and interconnectivity of the pores (Figure 2) (Collins and Birkinshaw, 2013; Echeverria Molina et al., 2021). Importantly the material itself but also its degradation products should be nontoxic. Additionally, the biomaterial surface should allow for modification with signalling molecules for the DCs and/or T cells (for some designs), allow for cell adhesion, promote cell growth, support cell functions, and finally, the material should have a good shelf life, and be reproducible (Collins and Birkinshaw, 2013; Echeverria Molina et al., 2021). Defining scaffold biocompatibility remains a topic of discussion (Williams, 2022), though a paper by Williams stated that a bioactive material should beneficially and appropriately direct interactions between the host system and the material through modulation of biological activity (Williams, 2022). Moreover, during the Chengdu conference in 2018 on definitions related to biomaterials, biocompatibility was defined as “the ability of a material to perform its desired functions with respect to a medical therapy, to induce an appropriate host response in a specific application and to interact with living systems without having any risk of injury, toxicity, or rejection by the immune system and undesirable or inappropriate local or system effects” (Ghasemi-Mobarakeh et al., 2019). Importantly, any interactions with the host system should be intentional, based on the design of the material and should not be a passive or accidental response, which requires that the interactions between the biomaterial and the host are well understood (Williams, 2022). However, often the host response against biomaterials remains largely unclear. A better understanding of these response mechanisms could have a significant impact on the clinical translation of biomaterials (Williams, 2022). Together, all above mentioned definitions underline that the biomaterial should have specific well understood immunomodulatory effects i.e., recruitment and stimulation of only DCs or T cells in the case of scaffolds for cancer therapy without inducing inflammation or toxicities.

**FIGURE 3:**
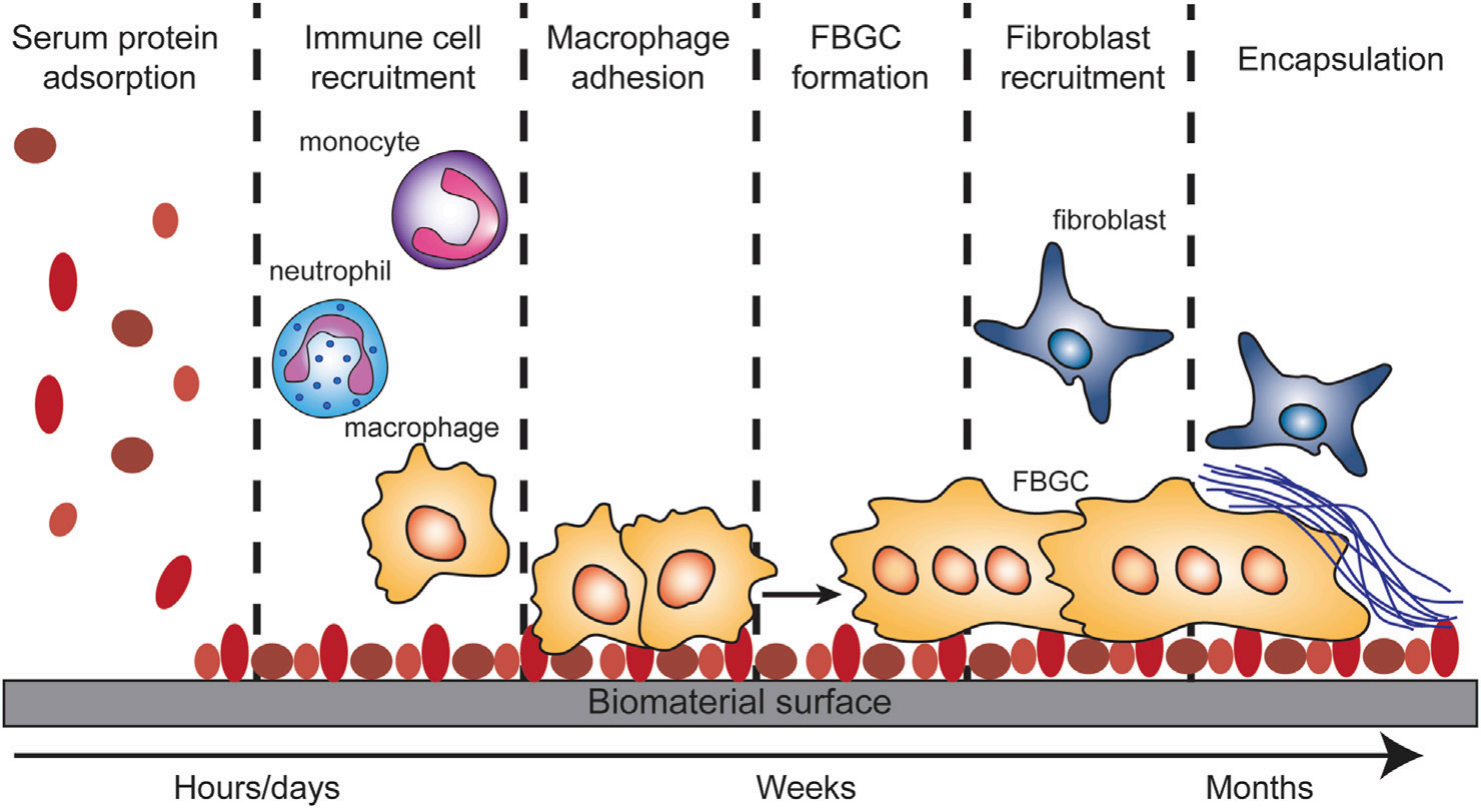
Foreign body response (FBR). The FBR is initiated by serum protein adsorption onto the biomaterial surface. This adsorption results in the recruitment of monocytes, neutrophils and macrophages. The macrophages will adhere to the protein covered surface and try to take up the biomaterial or to degrade it. When the biomaterial is too big for the macrophages to phagocytose, they will fuse into foreign body giant cells. The FBGC will recruit fibroblast which in turn will deposit matrix collagens on/around the biomaterial which results in the encapsulation of the biomaterial.

Numerous scaffolds have been designed for TE and tissue regeneration purposes (Memic et al., 2019; Echeverria Molina et al., 2021). The unwanted inflammatory response is one of the main challenges in the design of scaffolds for TE, which can lead to rejection of the scaffold by the surrounding tissue, in a process called the host response or foreign body response (FBR) (Figure 3) (Collins and Birkinshaw, 2013; Echeverria Molina et al., 2021; Whitaker et al., 2021). At the 2018 Chengdu conference, the host response or FBR was defined as “the cellular reaction of the biomaterial/tissue that is initiated by monocyte adhesion to the absorbed blood protein layer with subsequent monocyte differentiation to macrophage formation that may fuse to form foreign body giant cells (FBGC)” (Ghasemi-Mobarakeh et al., 2019). The FBR starts with oedema formation at the site of implantation or injection which leads to plasma protein accumulation (Kämmerling et al., 2021). The absorption of these proteins on the surface of the scaffold results in the formation of a chemotactic gradient for pro-inflammatory innate immune cells, such as neutrophils, macrophages, and monocytes (Whitaker et al., 2021). The recruited macrophages will make attempts to phagocytose the implant. If the implant is too large for phagocytosis, macrophages start producing reactive oxygen species (ROS) and enzymes to degrade the scaffold into smaller pieces which they can phagocytose (Kämmerling et al., 2021). Alternatively, the macrophages can fuse into FBGC at the surface of the implant (Christo et al., 2015), which also induces a phenotype switch from a pro-inflammatory M1-like phenotype to a wound-healing M2-like phenotype (Porcheray et al., 2005; Wu et al., 2020). The FBGC will recruit fibroblasts to the site of implantation, resulting in the deposition of matrix collagens and the formation of a fibrotic capsule (Anderson et al., 2008; Bridges et al., 2010). For some implants this fibrosis is beneficial, such as with surgical meshes which are intended to seal internal wounds (Baylón et al., 2017; Witherel et al., 2019). However, for most implant designs the formation of a fibrous capsule is detrimental for the intended purpose of the implant. Several of the parameters associated with the host response can be measured, such as fibrous capsule thickness, innate immune cell infiltration (especially neutrophils and macrophages) (Rostam et al., 2020), and serum levels of inflammatory markers (C reactive protein, liver markers, inflammatory cytokines) (Figure 2B) (Williams, 2008; Carnicer-Lombarte et al., 2021). We will use these parameters in the next sections to evaluate the host response towards biomaterial-based synthetic immune niches for cancer immunotherapy.

### Effect of scaffold-based cancer vaccine design on the host response

First, we investigated how scaffolds that actively recruit specific immune cells for *in vivo* immune activation affect the FBR (Scaffold-based cancer vaccines, Figure 1A and Table 1). Different strategies have been developed to incorporate recruitment factors into scaffold designs. To recruit DCs, numerous scaffold designs incorporate the cytokine granulocyte macrophage colony stimulating factor (GM-CSF) via physical encapsulation (Koshy et al., 2014; Bencherif et al., 2015; Shih et al., 2018; Bauleth‐Ramos et al., 2019), by using the anchoring capacities of gold nanoparticles (Verbeke and Mooney, 2015; Verbeke et al., 2017), by encapsulation in microspheres (Ali et al., 2009), or by adsorption on mesoporous silica rods (MSR) (Kim et al., 2015; Dellacherie et al., 2018; Li et al., 2018). CCL17 has also been physically encapsulated into scaffold walls to attract DCs (Zhan et al., 2017), while CCL21 has been loaded into the nanoparticles (NP) of polymer-nanoparticle (PNP) hydrogels (Fenton et al., 2019). Furthermore, microparticles have been used to recruit T regulatory cells to grafted tissue using CCL21 (Fisher et al., 2020). Other groups have used the chemokine-binding properties of heparin to incorporate chemokines into their scaffold design (Pérez del Río et al., 2020). Incorporation of cell lysates (Thelin et al., 2017) or antigens [such as the model antigen ovalbumin (OVA)] (Thelin et al., 2017; Kwee et al., 2019; Roth et al., 2020) was also shown to induce immune cell recruitment. However, whether these additions, especially OVA, can be called recruitment factors is debatable. The recruitment seen could also be due to other factors, e.g., endotoxins, which are introduced into the scaffold along with the antigen. In the case of T cell recruitment, recruitment could also be due to the uptake of the antigen by antigen-presenting cells (APCs) which in turn attract T cells.

**TABLE 1:** Examples of studies reporting cell infiltration in scaffolds actively recruiting immune cells. The table gives an overview of different materials used for scaffolds actively recruiting immune cells and provides details about the recruitment factor used, the infiltrating target and non-target cells, the mouse model used, and the delivery route tested. The cell numbers or percentages given in the table are the data that has been provided in the references or supplemental information and is the average per mouse.

**Biomaterial**	**Recruitment factor**	**Infiltration target cells**	**Infiltration non-target immune cells**	**Mouse model and outcome**	**Delivery route**	**Ref**
PLG matrices—measured d6, d14, d28	3 µg GM-CSF (encapsulated in PLG microspheres)	DCs (CD11c^+^CD86^+^): 30% (Total cells max: 2.3∙10^6^)	Not specified	Immunocompetent (C57BL/6J) GM-CSF incorporation resulted in increased DC recruitment. Addition of CpG-ODN and B16F10 lysates into scaffold vaccine design induced 90% survival in a prophylactic B16F10 melanoma model	Implantation s.c.	Ali et al. (2009)
	Blank	DCs (CD11c^+^CD86^+^): 14% (Total cells max: 0.6∙10^6^)				
Methacrylated gelatin cryogel—measured d14	Unmodified	Not specified	Total live cells: ∼0.1∙10^6^	Immunocompetent (C57BL/6J) GM-CSF modification increases cellular infiltration, capsule formation, and decreases degradation time	s.c. injection with 16G needle, 5 mm diameter and 2 mm thick in 200 µL Dulbecco’s PBS	Koshy et al. (2014)
	Encapsulated GM-CSF 5 µg	Not specified	Total live cells: ∼2∙10^6^			
Alginate hydrogel—measured d3, 5, 10, and 14	3 µg GM-CSF coupled to gold-NP	DCs (CD11b^+^CD11c^+^): max >4∙10^6^ (Total cells max: 5∙10^6^)	Macrophages and DC subset (F4/80^+^): max 2.2∙10^6^ Monocytes/granulocytes (Gr-1^+^): ∼2.5∙10^6^ NK/NK-T/T (DX5^+^): 5% d3, 25% d14	Immunocompetent (C57BL/6J) Scaffolds can be engineered to locally enrich immature DCs *in vivo*, especially by using GM-CSF coupled to gold-NP	Injection s.c. with 16G needle, 100 µL	Verbeke and Mooney (2015)
	3 µg GM-CSF free/soluble	DCs (CD11b^+^CD11c^+^): max 1.4∙10^6^ (Total cells max: 1.6∙10^6^)	Macrophages and DC subset (F4/80^+^): max 1.5∙10^6^ Monocytes/granulocytes (Gr-1^+^): limited (∼8-20%) NK/NK-T/T (DX5^+^): 10% d3 and d14			
	Blank	DCs (CD11b^+^CD11c^+^): very limited (Total cells: ∼0.1∙10^6^)	Macrophages and DC subset (F4/80^+^): max 0.2∙10^6^ Monocytes/granulocytes (Gr-1^+^): limited (∼8-20%) NK/NK-T/T (DX5^+^): 15% d3, 5% d14			
mesoporous silica rods – measured d7	1 µg GM-CSF (adsorbed onto MSR, including CpG and OVA)	DCs (CD11c^+^): 3∙10^6^ (Total cells: 25∙10^6^)	B cells (B220^+^): 21% T cells (CD3^+^): ∼2.5% NK cells (NK1.1^+^): ∼2.5% Monocytes (CD14^+^): 52%	Immunocompetent (C57BI/6J) Blank scaffolds were degraded within 25 days MSR vaccine resulted in enhanced IgG_1_ and IgG_2a_ serum levels and OVA specific CTL responses	Injection s.c. of 5 mg MSR in 150 µL PBS using 18G needle	Kim et al. (2015)
	Blank	DCs (CD11c^+^): 1∙10^6^ (Total cells: 9∙10^6^)	Not specified			
Alginate cryogel—measured d4	1.5 µg GM-CSF (physically encapsulated, including CpG ODN and irradiated F10-B16 cells)	DCs (CD11b^+^CD11c^+^): 3∙10^6^ (Total cell: 7∙10^6^)	Not specified	Immunocompetent (BALB/c and C57BL/6J) Alginate cryogel vaccine recruited high number of DCs and resulted in 80% overall survival of animals in a prophylactic B16F10 melanoma model with a rechallenge. In a therapeutic B16F10 melanoma model 40% of the mice survived until day 100	Injection s.c. of 2 cryogels with 16G needle	Bencherif et al. (2015)
	Blank	DCs (CD11b^+^CD11c^+^): 1∙10^6^ (Total cells: 2.5*10^6^)				
PLG scaffold—measured d14	β-cell lysates	T cells: 1.5% CD4^+^, 1.3% CD8^+^ (Total cells: not specified)	Macrophages (MAC1^+^): 49.4% DCs (CD11c^+^): 16.7% Granulocytes (Gr1^+^MAC1^+^): 17.4%	NOD mice (diabetic model) β-cell scaffolds enrich autoimmune T cells locally but do not affect diabetes development, can aid in the identification of autoimmune T cells	Implantation s.c. discs 1cm in diameter ± 0.2 cm width	Thelin et al. (2017)
	Blank	T cells: 1.1% CD4^+^, 0.5% CD8^+^ (Total cells: not specified)	Macrophages (MAC1^+^): 38.3% DCs (CD11c^+^): 40.6% Granulocytes (Gr1^+^MAC1^+^): 9.0 %			
Alginate hydrogel—measured d1, 3, and 5	GM-CSF coupled to gold-NP (with BDC peptide-loaded PLG microparticles)	DCs (CD11c^+^): max ∼25% (Total cells: not specified)	Macrophages (F4/80^+^): limited, max ∼10% Monocytes/granulocytes (GI-I^+^): all days ∼90%	Immunocompetent (C57BL/6J) Incorporation of peptide-loaded PLG particles is associated with significant differences in immune cell infiltration	Injection s.c. with 18G needle	Verbeke et al. (2017)
	GM-CSF coupled to gold-NP (without PLG microparticles)	DCs (CD11c^+^): max ∼80% (Total cells: not specified)	Macrophages (F4/80^+^): 20-60% Monocytes/granulocytes (GI-I^+^): max ∼80% d1, but drops to ∼5% on d5			
Alginate tough cryogel—measured d7	1.5 µg GM-CSF (physically encapsulated)	DCs (CD11b^+^CD11c^+^): 0.6∙10^6^ (Total cells 3∙10^6^)	Neutrophils (CD11b^+^Ly6G^+^): 0.2∙10^6^ Macrophages (CD11b^+^F4/80^+^): 0.1∙10^6^	Immunocompetent (BALB/c) Cryogel vaccine enhanced recruitment of DCs Addition of CpG-ODN and irradiated tumour cells (DD) to the cryogel vaccine design resulted 80% survival in a prophylactic murine breast cancer model	Injection s.c. of 2 cryogels with 16G needle	Shih et al. (2018)
	Blank	DCs (CD11b^+^CD11c^+^): 0.18∙10^6^ (Total cells 0.6∙10^6^)	Neutrophils (CD11b^+^Ly6G^+^): limited <0.1∙10^6^ Macrophages (CD11b^+^F4/80^+^): 0.6∙10^6^			
PNP hydrogel (HPMC-C_12_ PEG-PLA NP)—measured d7	100 µg OVA	Macrophages: 23.36% DCs: 20.48% (Total cells: 1∙10^6^)	Neutrophils: 12.86% Monocytes: 11.74% Other myeloid: 15.47% Non-myeloid: 13.09%	Immunocompetent (C57BL/6J) Vaccine loaded gels were able to recruit more immune cells and they were able to recruit APCs	Injection s.c. of 100 µL with a 21G needle	Roth et al. (2020)
	Blank	Macrophages: 32.54% DCs: 28.5% (Total cells: 0.2∙10^6^)	Neutrophils: 5.59% Monocytes: 1.89% Other myeloid: 12.1% Non-myeloid: 19.38%			

Some studies use the immunogenic properties of the scaffolds as adjuvants to enhance the immune response. This is for instance the case for MSRs described by Kim *et al.* (Kim et al., 2015). It was established that mesoporous silica can be degraded over time *in vivo* (Hudson et al., 2008), and the MSR described by Kim *et al.* was shown to degrade within 25 days without signs of toxicity or inflammation in liver, kidney, or other organs. However, high numbers of immune cells were observed to infiltrate the MSR scaffolds. The authors speculate that the NALP3 (NLRP3) inflammasome activation by silica and alternative complement activation lead to inflammation by MSR. They state that these immunostimulatory properties could be beneficial for the anti-tumour immune response and act as an additional adjuvant property of the scaffold. Even though this might be the case, the immunogenic properties might still lead to a fibrous capsule around the scaffold which could have a negative effect. Moreover, the immune cell recruitment and activation caused by the immunogenic properties of the scaffold itself might make it more difficult to induce a controlled and targeted immune response towards the DCs and T cells. This raises the question whether these MSR-based scaffolds are biocompatible when considering the definition given by Williams stating that the interaction between the scaffold and the host should be intentional, related to the design of the scaffold and not accidental (Williams, 2022). However, these inherent immunostimulatory properties of MSR could be exploited for specific scaffold designs, but still the question remains whether they can steer the design in such a way that they induce a controlled and targeted DC and T cell based immune response towards the tumour.

The incorporation of chemo-attractants into scaffolds increases the overall immune cell infiltration, and in most cases increases target cell recruitment/enrichment (Table 1). The number of total recruited immune cells differs largely between the scaffold designs, ranging from 0.2∙10^6^ to 9∙10^6^ for blank scaffolds and from 1∙10^6^ to 25∙10^6^ for scaffolds modified with recruitment factors. When the non-target cell infiltration in the scaffold is reported, the majority of these cells are either macrophages or neutrophils (Table 1). For some scaffold designs this recruitment is partly due to the material used, as is the case for the poly (lactic-co-glycolic) (PLG)-based scaffold, which by itself already has inflammatory properties which can attract cells from the myeloid lineage (Ali et al., 2009). Koshy *et al.* investigated the effect of GM-CSF on the biocompatibility, biodegradability and general cellular infiltration of gelatin cryogels (Koshy et al., 2014). They observed 20 times more living cells in GM-CSF containing scaffolds. Moreover, the GM-CSF containing scaffold also induced formation of a thick fibrous capsule which contained a large granulocytic cellular infiltrate. Additionally, they compared the degradation times for blank and GM-CSF modified scaffolds. The GM-CSF-modified cryogels, with the higher cell infiltrate and thicker fibrous capsule also degraded more rapidly over the course of 18 weeks compared to blank gelatin cryogels. Multiple other groups have also incorporated GM-CSF as a recruitment factor to recruit DCs (Bencherif et al., 2015; Kim et al., 2015; Verbeke and Mooney, 2015). These groups reported high DC recruitment, up to 3∙10^6^ DCs for the scaffolds with GM-CSF. These numbers seem extremely high, especially when considering that total DC numbers in a murine spleen have been reported to be around 2∙10^6^ (Kamath et al., 2000; McKenna et al., 2000; Kingston et al., 2009). GM-CSF is known to promote the development of monocyte derived DCs (moDCs) (Ushach and Zlotnik, 2016). This means that the monocytes attracted towards the scaffolds might have differentiated into moDCs, which could have contributed the high DC numbers observed by Kim *et al.* (Kim et al., 2015) and Bencherif *et al.* (Bencherif et al., 2015). Moreover, GM-CSF has a positive effect on macrophage and neutrophil recruitment (Ushach and Zlotnik, 2016), which could explain the large neutrophil and macrophage infiltration seen in all these studies. Interestingly, in another alginate cryogel study only 0.6∙10^6^ DCs were recruited using GM-CSF as recruitment factor (Shih et al., 2018), which would be more in line with the 2∙10^6^ DCs present in a complete mouse spleen.

Three of the scaffolds discussed in Table 1 tested their scaffold-based cancer vaccine in *in vivo* tumour models and reported promising anti-tumour immune responses (Ali et al., 2009; Bencherif et al., 2015; Shih et al., 2018). These three scaffolds either used tumour lysates or irradiated tumour cells as a source of tumour antigens and CpG-ODN as an adjuvant in addition to GM-CSF as a recruitment factor. Only Bencherif *et al.* showed tumour protection in a therapeutic model, when receiving two vaccinations 40% of the mice survived for up to 100 days (Bencherif et al., 2015). The other groups reported between 80% and 90% survival in a prophylactic setting (Ali et al., 2009; Shih et al., 2018).

Besides immune cell infiltration, the studies reported in Table 1 do not comment on fibrotic capsule formation or inflammatory molecules in the serum of these mice. Furthermore, not all studies report on the specific phenotype of the infiltrating non-target cells. In general, a more detailed investigation of immune cell infiltration, fibrotic capsule formation, and serum levels of inflammatory molecules in existing/reported scaffold-based vaccine systems are required to get a comprehensive overview of the extent of myeloid cell infiltration and biocompatibility across different scaffold types and setups.

### Host response towards scaffolds for ACT of CAR-T cells

In addition to scaffolds that actively recruit immune cells, scaffolds can also serve as protective environments during injection of cells for ACT (Scaffolds for ACT, Figure 1B), thereby preventing cell damage, ensuring local delivery, and especially local retention of cells (Mooney and Vandenburgh, 2008; Li et al., 2014; Amer et al., 2015). Moreover, biomaterials can be crucial to maintain the functional phenotype and viability of the encapsulated cells (Orive et al., 2003; Dawson et al., 2008). For example, it has been suggested that delivery using scaffolds is beneficial for the viability, local retention, and expansion of transferred stem cells (Foster et al., 2017; Grosskopf et al., 2020; Correa et al., 2021). Additionally, the scaffolds provide a local environment for continued T cell stimulation. Various scaffolds have been designed to aid in the ACT of CAR-T cells to induce anti-tumour responses (Stephan et al., 2015; Smith et al., 2017; Coon et al., 2020; Wang et al., 2020; Hu et al., 2021; Agarwalla et al., 2022; Grosskopf et al., 2022) (Table 2). All scaffold designs discussed in Table 2 show promising results concerning the anti-tumour immune responses, even leading to complete remission and protection against tumour recurrence in certain cases. Interestingly, the scaffolds differ largely in their design, ranging from large (700 µL) to small (1 µL), encapsulating high (10∙10^6^) to low (0.4∙10^6^) numbers of CAR-T cells. Moreover, the biomaterials used to produce the scaffold also differ between the individual designs (alginate, nitinol, chitosan, HA, and PNP hydrogel). Additionally, the chitosan-PEG hydrogel was only used as a depot for the CAR-T cells to ensure local delivery and persistence without any modification to incorporate T cell stimulatory molecules (Wang et al., 2020). The alginate cryogel described by Agarwalla *et al.* on the other hand used their scaffold to *in vivo* generate CAR-T cells with limited *ex vivo* manipulation of the T cells (Agarwalla et al., 2022).

**TABLE 2:** Overview of biomaterial-based scaffolds for ACT of T cells for cancer immunotherapy with tumour models. The table gives an overview of different materials used for ACT of T cells. The table proved details about the modifications of the scaffolds, the size of the scaffold, the numbers added per scaffold, the delivery route tested, and the mouse model tested.

Biomaterial	Modification	Size	Cells for *in vivo* experiments	Delivery	Mouse model and outcome	Ref
Macroporous alginate scaffold	GFOGER, stimulatory microspheres (αCD3, αCD28, αCD137 and IL-15SA)	700 µL, 15 mm round, 2 mm thick	7∙10^6^ murine 4T1-specific CD8^+^ T cells or 7∙10^6^ NKG2D murine CAR-T cells	Implanted in peritoneal or resection cavity	4T1 resection model (BALB/c) → protected from relapse Advanced-stage ovarian tumour model (Albino B6) → clearance in 6/10	Stephan et al. (2015)
Macroporous alginate scaffold	GFOGER, stimulatory microspheres (αCD3, αCD28, αCD137 and STING agonist (cdCMP))	700 µL, 15 mm round, 2 mm thick	7∙10^6^ murine CAR-T cells	Implanted peritoneal or resection cavity	KPC (Albino B6) → clearance in 4/10, all 4 mice rejected tumour rechallenge B16F10 resection model (CD45.1) → clearance in 6/10 mice, 6 rejected tumour rechallenge	Smith et al. (2017)
Nitinol thin films (2D films or 3D stents)	Fibrin coating, αCD3, αCD28, and αCD137	Film: 7 x 4mm^2^ Stent: 4 mm long, 4.5 mm diameter	Film: 10∙10^6^ human CAR-T cells Stents: 1.6∙10^6^ human CAR-T cells	Implanted next to the tumour (diaphragm or s.c.)	Film: OVCAR-3 (NSG) → clearance in 7/10, average survival 80d Stent: ROR1^+^PANC resection model (NSG) → CAR-T cell stents remained open lumen	Coon et al. (2020)
Chitosan-PEG hydrogel	none	1 µL, *in situ* gelation	1∙10^6^ human GD2(IL-15)-CAR-T cells	Injected under the retina	RB xenograft model (NU/NU nude mice) → controlled tumour growth and prevented tumour recurrence	Wang et al. (2020)
Hyaluronic acid hydrogel	Platelets coated with αPDL1 (1ug) and NP with IL-15 (1ug)	48 well, 400 µL	2∙10^6^ human CSPG4 CAR-T cells	Implanted in resection cavity (s.c.)	WM115 (NSG) human melanoma resection model → smallest tumour signal, CAR-T cell persistence up to 4 weeks Double-tumour model → abscopal effect, inhibition of contralateral tumour	Hu et al. (2021)
Alginate cryogel	αCD3, αCD28, and IL-2	48 well with 300 µL	1∙10^6^ human PBMCs (20% is transduced, 0.2∙10^6^ CAR-T cells) and virus particles (total 2∙10^6^ PBMCs)	Implanted s.c., 2 scaffolds per mouse	CD19 model (Daudi cells) → tumour free survival of 50% d100	Agarwalla et al. (2022)
PNP hydrogel (HPMC-C_12_ and PEG-PLA NP)	RGD, IL-15 (encapsulated in the NP)	100 µL, *in situ* gelation	2∙10^6^ B7H3 human CAR-T cells	Injected s.c. peritumour, 21G needle	MED8A solid tumour (NSG) → complete clearance, distant tumour could be cleared within 30d	Grosskopf et al. (2022)

Besides these differences in design, the majority of these scaffolds are delivered via implantation (Stephan et al., 2015; Smith et al., 2017; Coon et al., 2020; Hu et al., 2021; Agarwalla et al., 2022). As a minimally invasive alternative, two injectable hydrogels have been described for the local delivery of CAR-T cells. Wang *et al.* described the use of small (1 µL) injectable chitosan-PEG hydrogel (Wang et al., 2020) and Grosskopf *et al.* engineered an injectable PNP hydrogel (Grosskopf et al., 2022). Additionally, the majority of the reported scaffolds provided CAR-T cells with stimulatory antibodies (αCD3, αCD28, and αCD137), cytokines (IL-15(SA) or IL-2) and cell-adhesive molecules (GFOGER, fibrin or RGD) (Table 2). With the exception of the nitinol-based scaffold (Coon et al., 2020), all reports used IL-15 or IL-2 in their design, although Wang *et al.* included the IL-15 in the CAR-T cell genetic construct and not in their scaffold design (Wang et al., 2020). The addition of the cytokines provided T cells with additional survival and stimulatory signals, resulting in improved anti-tumour immune responses compared to the designs without cytokines. Incorporation of cytokines in the scaffold design might enhance the normally short half-live of cytokines (Lotze et al., 1985). Additionally, it prevents systemic exposure to high doses of cytokines, which could lead to capillary leak syndrome and multiple organ failure (Rosenberg, 2014; Weber et al., 2015; Jiang et al., 2016). The addition of cell-adhesive molecules into the reported scaffold designs resulted in increased T cell motility and improved viability (Stephan et al., 2015; Smith et al., 2017; Grosskopf et al., 2022). All these reported scaffold design options indicate the versatility of biomaterial-based scaffolds for aiding ACT (CAR-)T cells and underline the positive effects of local delivery of (CAR-) T cells on tumour clearance.

Of these studies, only three reported on the biocompatibility of their scaffolds in terms of fibrotic capsule formation, host immune cell infiltration or serum levels of inflammatory molecules (Table 3) (Coon et al., 2020; Agarwalla et al., 2022; Grosskopf et al., 2022). Most of the CAR-T cell studies perform their functional read-out (anti-tumour immunity) in immunodeficient NSG mouse models. It is therefore important to emphasise that a study performed by Kerr *et al.* clearly showed differences in HA scaffold half-life and immune cell infiltration between immunocompetent mice (C57BL/6J) and NSG mice (Kerr et al., 2022). Comparison of scaffold stability indicated a half-life of about 9.5 days in immunocompetent mice, while in NSG mice a reduction of only 35% was observed after 3 months (Kerr et al., 2022). Furthermore, significant differences in myeloid cell infiltration were observed: infiltration of around 7∙10^5^ myeloid cells on day 10 for the C57BL/6J immunocompetent mice compared to 0.5∙10^5^ for the NSG mice. The majority of the infiltrated myeloid cells were neutrophils (CD11b^+^F4/80^-^Ly6G^+^) (>80%) for the C57BL/6J immunocompetent mice. The NSG mice showed high levels of neutrophils on day 1 (90%) which changed to mainly macrophages (85%) by day 10 (Kerr et al., 2022). In addition, the mouse strain used can also affect the fibrotic response towards the implanted biomaterial (King et al., 2001). It is for instance known that the C57BL/6J mouse strain has a more Th1-prone immune response, while the BABL/c mouse strain has a more Th2-prone immune response (Trunova et al., 2011; Fornefett et al., 2018). Moreover, it has been shown that implantation of alginate microspheres in the peritoneal cavity of BALB/c mice resulted in limited fibrotic capsule formation (King et al., 2001), while a large fibrotic overgrowth was observed when the alginate microcapsules were implanted in C57BL/6J mice (King et al., 2001; Spasojevic et al., 2014; Vegas et al., 2016).

**TABLE 3:** Overview of studies reporting immune cell infiltration using biomaterial-based scaffolds for (CAR-)T cell adoptive cell transfer. The table gives an overview of different materials used for ACT of T cells and provides details about the mouse model used, the presence of a fibrotic capsule, the immune cell infiltration, long term stability and biocompatibility, and the delivery route tested.

Biomaterial	Mouse model	Fibrotic capsule	Immune cell infiltration	Long term stability	Long term biocompatibility	Delivery route	Ref
HA cryogel—1, 5, and 10 days	Immunocompetent (C57BL/6J)	N.A.	Day 10, mainly neutrophils (>80%, 6.5∙10^6^ cells), some macrophages (<5%, 2.5∙10^4^)	Short, average half-life 9.5 days	N.A.	Injection s.c. with 16G needle	Kerr et al. (2022)
	Immunodeficient (NSG)	N.A.	Day 10, mainly macrophages (90%, 3∙10^4^), very little neutrophils	Long, average half-life >3 months			
Nitinol film—4 months	Female NSG	Thin layer—4 months	Limited (macrophages, lymphocytes and multinucleate giant cells)	Yes, material is non-degradable	Yes, no changes in ALT, AST, LDH or CRE	Implanted	Coon et al. (2020)
Alginate Histopathology at 4 weeks Immune cell infiltration at day 4	Immune competent (C57BL/6J) for histopathology study. NSG mouse engrafted with human PBMCs for immune cell infiltration study	Thin layer—4 weeks	Limited at day 4, mainly murine CD11b^+^ (80% of all infiltrating cells)	Yes, material is non-degradable	Yes, no histopathology of the major organs at 4 weeks. Blood biochemical analysis was fine	Implanted	Agarwalla et al. (2022)
PNP hydrogel—4 weeks	Immune competent (SKH1-Elite), for biocompatibility study	No visible sign	N.A.	Short, retention half-life of 8.9 ± 2.6 days	N.A.	Injected s.c. 21G needle	Grosskopf et al. (2022)

Of the three CAR-T cell studies discussed in Table 3 Agarwalla *et al.* (Agarwalla et al., 2022) and Grosskopf *et al.* (Grosskopf et al., 2022) performed separate biocompatibility experiments using immunocompetent mice. Coon *et al.* solely reported on the biocompatibility of their scaffold design in immunodeficient NSG mice (Coon et al., 2020). All three ACT studies reported either no visible sign of a fibrotic capsule, or only a thin layer. In addition, Agarwalla *et al.* (Agarwalla et al., 2022) and Coon *et al.* (Coon et al., 2020) also investigated the immune cell infiltration, reporting only limited immune cell infiltration, of which the majority were myeloid derived CD11b^+^ cells.

### Effect of scaffold surface modification on biocompatibility

There are many different possibilities to modify scaffolds, ranging from chemically modifying the polymers that form the basis of the scaffolds to coatings applied after scaffold production. Besides modifying the scaffold to direct the behaviour and functionality of the ‘target cells’, modifications can also greatly impact biocompatibility, such as fibrotic capsule formation and unwanted immune cell infiltration (Table 4) (Echeverria Molina et al., 2021; Whitaker et al., 2021). Unfortunately, the effect of some modifications on fibrous capsule thickness has shown varied outcomes, which complicates design recommendations.

**TABLE 4:** Examples of biomaterial modification and their influence on the immune cell response and fibrous capsule formation. The table gives an overview of different materials used for scaffold designs and provides details about the modifications, the infiltrating immune cells, the size of the fibrous capsule, the mouse model used, and the delivery route tested. The cell numbers or percentages given in the table are the data that has been provided in the references or supplemental information and is the average per mouse.

Biomaterial	Modification	Infiltration immune cells	Fibrous capsule	Mouse model and outcome	Delivery route	Ref
Silicon wafer coated with 25 nm gold	Unmodified	Total cells: 2∙10^6^ Activated fibroblasts (Mac-1^+^): 5∙10^5^	110 µm	BALB/c immunocompetent -CH_3_ modification results in thicker fibrous capsule. Both -OH and -CH_3_ modification results in increased immune cell infiltration (especially activated fibroblasts)	Implantation in s.c. air pouch cavity, implant 0.5 x 0.5 cm^2^	Barbosa et al. (2006)
	-OH	Total cells: 3∙10^6^ Activated fibroblasts (Mac-1^+^): 1.8∙10^6^	70 µm			
	-CH_3_	Total cells: 3∙10^6^ Activated fibroblasts (Mac-1^+^): 2.2∙10^6^	120 µm			
	-COOH	Total cells: 2∙10^6^ Activated fibroblasts (Mac-1^+^): 1∙10^6^	80 µm			
Polypropylene microspheres—measured after 2 weeks	Unmodified (-CH_2_)	CD11b^+^ inflammatory cells: 125.3 ± 31.2/mm^2^	65.1 ± 10.3 µm With high collagen deposition	BALB/c immunocompetent Density of the chemical modification had only minor effects on the FBR. -OH resulted in increased capsule and immune cell infiltration while -COOH reduced the capsule formation and immune cell infiltration	Injected s.c. with 18G needle, 100 mg particles (35 µm diameter) in 0.5 mL saline	Nair et al. (2008)
	-OH (low (L), medium (M), and high (H) densities)	CD11b^+^ inflammatory cells: L: 366.3 ± 113.6/mm^2^ M: 326.3 ± 48.5/mm^2^ H: 322.6 ± 44.9/mm^2^	L: 134.4 ± 27.5 µm M: 109.2 ± 10.0 µm H: 101.8 ± 21.4 µm With high collagen deposition			
	-COOH (low (L), medium (M), and high (H) densities)	CD11b^+^ inflammatory cells: L: 79.0 ± 14.0/mm^2^ M: 56.2 ± 10.7/mm^2^ H: 62.1 ±16.4/mm^2^	L: 30.0 ± 2.2 µm M: 37.0 ± 10.2 µm H: 37.4 ± 6.1 µm With minimal collagen deposition			
Polypropylene microspheres—measured after 2 weeks	Unmodified	CD11b^+^ inflammatory cells: 26.25 ± 8.13/mm^2^	55.3 ± 10.5 µm	BALB/c immunocompetent The chemical nature of the surface of s.c. implanted scaffolds modulate capsule thickness, cell infiltration depth, and cell number. With the -COOH modification resulting in lowest immune response	Implanted s.c., 35 µm diameter	Kamath et al. (2008)
	-OH	CD11b^+^ inflammatory cells: 135.25 ± 36.86/mm^2^	Highest thickness, 251 ± 45.6 µm			
	-NH_2_	CD11b^+^ inflammatory cells: 261 ± 39.35/mm^2^	151.7 ± 35.3 µm			
	-CF_x_	CD11b^+^ inflammatory cells: 50.25 ± 12.03/mm^2^	101.3 ± 35.9 µm			
	-COOH	CD11b^+^ inflammatory cells: 11.75 ± 1.5/mm^2^	23.4 ± 2.8 µm			
Mesoporous silica microrod scaffold—measured day 5	Unmodified	Total cells 1.4∙10^6^, myeloid/neutrophils (Ly6G^High^Ly6C^Mid^) 62%	∼30 mg	Immunocompetent (C57BL/6J) PEG modification increased immune cell infiltration and capsule formation, PEG-RGD modification resulted in less immune cells and capsule compared to PEG only	5 mg MSR injected s.c. in 150 µL PBS using 18G needle	Li et al. (2016)
	PEG	Total cells 1.4∙10^7^, myeloid/neutrophils (Ly6G^High^Ly6C^Mid^) 78%	Heavier (∼95 mg), thicker and higher levels of IL-1β compared to unmodified			
	PEG-RGD	Total cells 2.5∙10^6^, myeloid/neutrophils (Ly6G^High^Ly6C^Mid^) 55%	Similar to unmodified (∼40 mg)			
Mesoporous silica microrod scaffold—measured d3	Unmodified (including 100 µg CpG, 50 µg OVA, 1 µg GM-CSF)	Total cells: 0.9∙10^6^ Activated DCs (CD11c^+^CD86^+^/CCR7^+^): 0.062∙10^6^	Not mentioned	Immunocompetent (C57BL6J) PEI modification did not increase total immune cell infiltration but did increase the number of recruited activated DCs	5 mg MSR injected s.c. in 150 µL PBS using 18G needle	Li et al. (2018)
	PEI modified (including 100 µg CpG, 50 µg OVA, 1 µg GM-CSF)	Total cells: 1∙10^6^ Activated DCs (CD11c^+^CD86^+^/CCR7^+^): 0.135∙10^6^				
Silicon wafers alginate layer—measured after 1 month	PLL_100_ coated	Not quantified, high numbers of macrophages and fibroblast were found around the implants. 97.25 ± 5.5% of the implants had cellular overgrowth	Implants formed clumps (sticking to abdominal organs) and were caught in thick layers of fibroconnective tissue	Immunocompetent (Balb/c) Addition of the PEG-_454_-b-PLL_50_ diblock copolymer reduced the host immune response against alginate-PLL_100_	Injected with 16G needle via incision in the peritoneal cavity, at least 1000 capsules in 0.5 mL	Spasojevic et al. (2014)
	PLL_100_-PEG_454_-b-PLL_50_ coated	Not quantified, 36.25 ± 27.8% of the implants had cellular overgrowth. Mostly, just a few cells, which were mainly macrophages and a few fibroblasts	Implants did not form clumps and no sticking to abdominal organs was observed			
Alginate microcapsule (hydrogel)—measured day 14	Unmodified	Macrophages (CD68^+^CD11b^+^): 14∙10^4^ per 100 µL retrieved capsule Neutrophils (Ly6G^+^CD11b^+^): 2.6∙10^4^ per 100 µL retrieved capsule	Thicker fibrous deposition ∼0.045 ng collagen/sphere	Immunocompetent (C57BL/6J) The chemical modifications, all containing a triazole functionality, showed a lack of immune cell recruitment and activation on the surface. Moreover, limited fibrous deposition was observed	Implanted in the peritoneal cavity, diameter of 300-350 µm, 350 µL in total	Vegas et al. (2016)
	Triazole functionalized (Z2-Y12, Supplementary Figure S1)	Macrophages (CD68^+^CD11b^+^): ∼0.5∙10^4^ per 100 µL retrieved capsule Neutrophils (Ly6G^+^CD11b^+^): ∼0.01∙10^4^ per 100 µL retrieved capsule	Almost no fibrous deposition ∼0.018 ng collagen/sphere			
	Triazole functionalized (Z1-Y15, Supplementary Figure S1)	Macrophages (CD68^+^CD11b^+^): 2∙10^4^ per 100 µL retrieved capsule Neutrophils (Ly6G^+^CD11b^+^): ∼0.1∙10^4^ per 100 µL retrieved capsule	Almost no fibrous deposition ∼0.018 ng collagen/sphere			
	Triazole functionalized (Z1-Y19, Supplementary Figure S1)	Macrophages (CD68^+^CD11b^+^): 2∙10^4^ per 100 µL retrieved capsule Neutrophils (Ly6G^+^CD11b^+^): ∼0.15∙10^4^ per 100 µL retrieved capsule	Almost no fibrous deposition ∼0.02 ng collagen/sphere			
Agarose hydrogel or agarose/HA composite hydrogel—measured at week 1, 2, 3, 4, 5, 6, 8, 10, and 13	Agarose	Not quantified, a lot of macrophages and fibroblasts	Slight fibrous capsule	Immunocompetent (specific pathogen free Kunming) Composite hydrogel showed more rapid but lesser inflammatory response and improved degradation	s.c. implantation	Zhang et al. (2012)
	Agarose/HA composite	Not quantified, slight capsule of fibroblasts and macrophages	Slight capsule formation			
Clinical grade silicon rubber coated with meth(acrylate) and meth(acrylamide) monomers—measured at 28 days	No coating	Macrophages: 70 per cm^2^ Neutrophils: 10 per cm^2^	Collagen thickness around 40 µm	Immunocompetent (Balb/c) M2 coating resulted in least cells but the M0 coating resulted in least amount of collagen, indicating that the presence of both M1 and M2 (M0 coating) reduced fibrotic tissue formation.	s.c. implantation	Rostam et al. (2020)
	M0 coating (C398 or C408)	Macrophages: ∼50 per cm^2^ Neutrophils: ∼5 per cm^2^	Collagen thickness ∼20 µm			
	M1 coating (H24 or C170)	Macrophages: ∼50per cm^2^ Neutrophils: ∼6 per cm^2^	Collagen thickness ∼30 µm			
	M2 coating (C255 and C301)	Macrophages: ∼30 per cm^2^ Neutrophils: ∼3 per cm^2^	Collagen thickness ∼40 µm			
Alginate microcapsule (hydrogel)—measured day 14	Triazole functionalized (Z1-A3, Supplementary Figure S1)	Showed less macrophage intensity (CD68) and less myofibroblast intensity (αSMA) compared to the control	Lower expression of αSMA and Colla1 indicating lower fibrosis and reduced collagen deposition	Immunocompetent (C57BL/6J) The hydrophilic PEG-linker-based small molecule leads work better when attached to hydrophilic alginates for cell encapsulation.	s.c. implantation	Mukherjee et al. (2022)
	Triazole functionalized (Z4-A10, Supplementary Figure S1)	Showed less macrophage intensity (CD68) and less myofibroblast intensity (αSMA) compared to the control	Lower expression of αSMA and Colla1 indicating lower fibrosis and reduced collagen deposition			
Medical grade silicone catheters—measured after 4 weeks	Unmodified		Fibrous capsule thickness: 125 µm	Immunocompetent (C57BL/6J) The hydrophobic lead showed better results when used to coat hydrophobic silicone catheters.	s.c. implantation	
	Methacryloyl modified Z1-A3 (Supplementary Figure S1)		Fibrous capsule thickness: 50 µm			
	Methacryloyl modified B2-A17 (Supplementary Figure S1)		Fibrous capsule thickness: 40 µm			

Modification of polypropylene with a hydroxyl group (-OH) resulted in higher levels of immune cell infiltration and thick fibrous capsules, while modification with a carboxylic acid group (-COOH) resulted in very limited immune cell infiltration and a thin fibrous capsule (Kamath et al., 2008; Nair et al., 2008). The density of the individual chemical groups only had a minor effect on the FBR (Nair et al., 2008). Interestingly, modification of silicon coated with gold with hydroxyl (-OH) groups resulted in the thinnest fibrous capsule, though the immune cell infiltration was elevated compared to the unmodified scaffold (Barbosa et al., 2006). A study by Li *et al.* investigated the effect of functionalization of MSR with poly (ethyleneglycol) (PEG) and the integrin-binding ligand RGD on immune cell activation and infiltration (Li et al., 2016). The authors expected to see reduced immune cell infiltration with the PEG modification, as PEG is considered nontoxic and nonimmunogenic, and increased immune cell infiltration with PEG-RGD, as RGD is widely used to enhance cell adhesion. Interestingly, the PEG modification resulted in nearly 10 times more immune cell infiltration compared to blank scaffolds, especially consisting of myeloid cells/neutrophils. PEG-RGD modification also increased the total immune cell infiltration compared to blank scaffolds but to a much lesser extent than the PEG modified scaffold. Additionally, the PEG modified scaffold gave rise to a thicker and heavier fibrous capsule compared to the blank and PEG-RGD. In another study by Li *et al.* the MSR was modified with polyethyleneimine (PEI) to enhance immunogenicity (Li et al., 2018). Both PEI-modified and blank scaffolds recruited similar numbers of total immune cells, though the PEI-modified scaffolds were able to more strongly enrich activated DCs. However, less than 1% of the total infiltrated immune cells proved to be activated DCs. Unfortunately, the composition of the other 99% of the infiltrating immune cells and the formation of a fibrous capsule were not discussed. A widely used polymer for scaffolds is the FDA approved natural algae-derived polymer alginate (Bencherif et al., 2012). Studies in humans, non-human primates and certain rodent strains, however, have indicated a FBR elicited against alginate implants (King et al., 2001; Jacobs-Tulleneers-Thevissen et al., 2013; Scharp and Marchetti, 2014). Due to the low production cost, tunability, and mild gelation (Lee and Mooney, 2012), there is an interest in investigating modifications to better control the FBR towards alginate. Modification of alginate gels with poly-L-Lysine (PLL)_100_ by Spasojeciv *et al.* resulted in a strong immune response directed against the alginate scaffold (Spasojevic et al., 2014). Addition of the di-block copolymer PEG-_454_-b-PLL_50_ to the PLL_100_ coated alginate scaffolds diminished this immune response to such an extent that limited cellular overgrowth was observed. Vegas *et al.* created an alginate modification library where they tested the FBR towards 634 different alginate modifications (Vegas et al., 2016). Three of the modifications, all containing a triazole functionality, displayed limited fibrous deposition and minimal macrophage and neutrophil recruitment. In addition to peritoneal implantation of the alginate modified scaffolds, Vegas *et al.* also implanted the modified alginate scaffolds subcutaneous (s.c.) where they observed lower cathepsin activity (a marker for immune cell activation), lower fibrotic overgrowth, and lower collagen levels at the implant surface compared to unmodified alginate 28 days post implantation (Vegas et al., 2016). In a follow-up study *Vegas et al.* created a new alginate-modification library based on the three triazole functionalities (Mukherjee et al., 2022). Besides modification of the polymers that make up the scaffolds, some groups combine different polymers to make composite materials to improve the FBR. Zhang *et al.* added hyaluronic acid to an agarose hydrogel which showed improved degradation kinetics and reduced infiltration by macrophages and fibroblasts compared to the agarose hydrogel (Zhang et al., 2012). This suggests that hyaluronic acid can be used in a composite material to improve the FBR. These studies indicate that unwanted infiltration of myeloid cells is a universal problem, though scaffold modification or composites can improve the FBR.

In addition to scaffold modification, the choice of polymer used for the scaffold can have a profound effect on the macrophage phenotype and capsule formation (Rostam et al., 2020). Moreover, scaffold size can influence the fibrous capsule formation to some extent, with larger scaffolds inducing thinner fibrotic capsules (Whitaker et al., 2021). However, size is not the only determining factor, as scaffold shape has also been suggested to influence the FBR. Spherically-shaped implants with smooth contours have shown improved resistance against fibrosis (Nair and Tang, 2017; Echeverria Molina et al., 2021). Moreover, pore size has also been reported to influence macrophage phenotype and fibrotic capsule formation. Relatively large pores (5–100 µm) induce higher tissue integration and less fibrotic capsule formation (Echeverria Molina et al., 2021; Whitaker et al., 2021). Finally, microstructure, coatings, topography, and degradation speed/time can have an effect on the FBR (Nair and Tang, 2017; Echeverria Molina et al., 2021; Whitaker et al., 2021; Williams, 2022).

## Discussion and future directions for the optimal design of biomaterial-based synthetic immune niches for cancer therapy

When designing a scaffold for immunotherapy there are certain parameters which are favourable for DC and T cell performance. First, an injectable scaffold allows for a minimally invasive delivery method circumventing risks associated with surgical implantation. Next, the degradation of the biomaterial should be in line with the optimal treatment time (Echeverria Molina et al., 2021). Too fast degradation might result in suboptimal treatment, while too slow degradation might result in undesired immune responses at the injection site. In the case of immunotherapy, a biodegradable scaffold is favourable over non-degradable materials, as this obviates the need for surgical removal of the scaffold at the end of the treatment. Therefore, it is advised to study long-term scaffold stability and to investigate the parameters influencing the degradation kinetics to design a scaffold with an optimal functional half-life. Moreover, scaffolds provide a local stimulatory immune niche, therefore the ideal location for scaffold administration will need to be determined. Shih *et al.* showed that cancer vaccine cryogels implanted further from the draining lymph node (dLN) induced less anti-tumour protection compared to cryogels injected close to the dLN (Shih et al., 2018). Indicating that injection near a lymph node might be most optimal for the cancer vaccine scaffolds but might also be interesting to consider for the ACT scaffolds. The scaffolds for ACT of CAR-T cells discussed in Table 2 were all injected/implanted in close proximity to the tumour or in the tumour resection area (Stephan et al., 2015; Smith et al., 2017; Coon et al., 2020; Wang et al., 2020; Hu et al., 2021; Agarwalla et al., 2022; Grosskopf et al., 2022). Even though scaffolds for ACT were implanted locally, Hu *et al.* demonstrated that they cause an abscopal effect, where their HA hydrogel provided anti-tumour effects towards the contralateral tumour (Hu et al., 2021). This indicates that even though the scaffolds deliver and sustain the CAR-T cells locally, they can also induce systemic tumour protection.

Additionally, the scaffolds should induce limited intrinsic host responses, while supporting the specific stimulation, survival, and persistence of the transferred CAR-T cells for ACT scaffolds and specific recruitment and activation of DCs for the cancer vaccine scaffolds. Therefore, more in-depth studies into the infiltration of (non-specific) immune cells and the formation of a fibrotic capsule are necessary. Importantly, the study by Kerr *et al.* clearly indicated a difference in FBR between immunocompetent and NSG mice (Kerr et al., 2022). Additionally, the effect of depletion of neutrophils, macrophages, B cells, and T cells was investigated in this study. To deplete macrophages, clodronate liposomes were administered intraperitoneally with 80%–95% efficiency. Interestingly, this high degree of macrophage depletion had a minimal effect on the macrophage infiltration in the HA cryogels. This indicates that depletion of peripheral blood monocytes by clodronate is not enough to affect the local, probably tissue resident, macrophage response towards s. c. injected scaffolds. Depletion of the neutrophils, B cells, and T cells did not influence the scaffold half-life. Neutrophil depletion did reduce both myeloid infiltration and neutrophil infiltration (Kerr et al., 2022).

In addition, the choice of recruitment and stimulatory signals is imperative to elicit a proper anti-tumour T cell-based immune response. The majority of the studies discussed in Table 1 used GM-CSF as a recruitment factor for DCs. The addition of GM-CSF to the scaffold design, however, did not only increase DC specific recruitment but also resulted in high influx of other myeloid immune cells, such as macrophages and neutrophils. To create a robust and specific immune response with these cancer vaccine scaffolds, a more specific recruitment factor should be investigated. Most of the studies discussed in Table 2 included at least a cytokine (IL-15 or IL-2) as an T cell stimulatory signal into their ACT scaffold design. Besides cytokines, some of the ACT scaffold designs also included stimulatory antibodies targeting the co-stimulatory receptor CD137 in addition to CD28 (Stephan et al., 2015; Smith et al., 2017; Coon et al., 2020). The versatility of the platforms also underlines the possibility to test additional parameters, like ligand spacing, ligand combinations, linker length, and substrate flexibility on the activation and phenotype of T cells. Though some groups have made initial steps to investigate these parameters, additional research is needed to determine the optimal scaffold design for T cell activation. The addition of different biomolecules is hypothesized to improve the anti-tumour immune response, although this also complicates the scaffold design. This increased complexity makes the characterization of the end-product more difficult, causes challenges for well-controlled scale-up potential, and convolutes the regulatory process for future clinical use. Moreover, production of clinical grade scaffolds according to GMP-compliant standards might present additional difficulties.

Besides the addition of stimulatory signals for the DCs or T cells, chemical modifications of the scaffolds could further improve the function of synthetic immune niched by reducing the unwanted host response. The studies discussed in Table 4 indicate that certain chemical modification might result in reduced host response towards scaffolds. It would therefore be interesting to add for instance the triazole functionality discussed by Vegas *et al.* to a scaffold design to improve the FBR, which was thus far only tested for alginate scaffolds. Overall, more research is needed into the effect of the modifications on the specific immune response and into the applicability of the modifications over a range of biomaterials used for scaffolds.

## Conclusion

**FIGURE 4:**
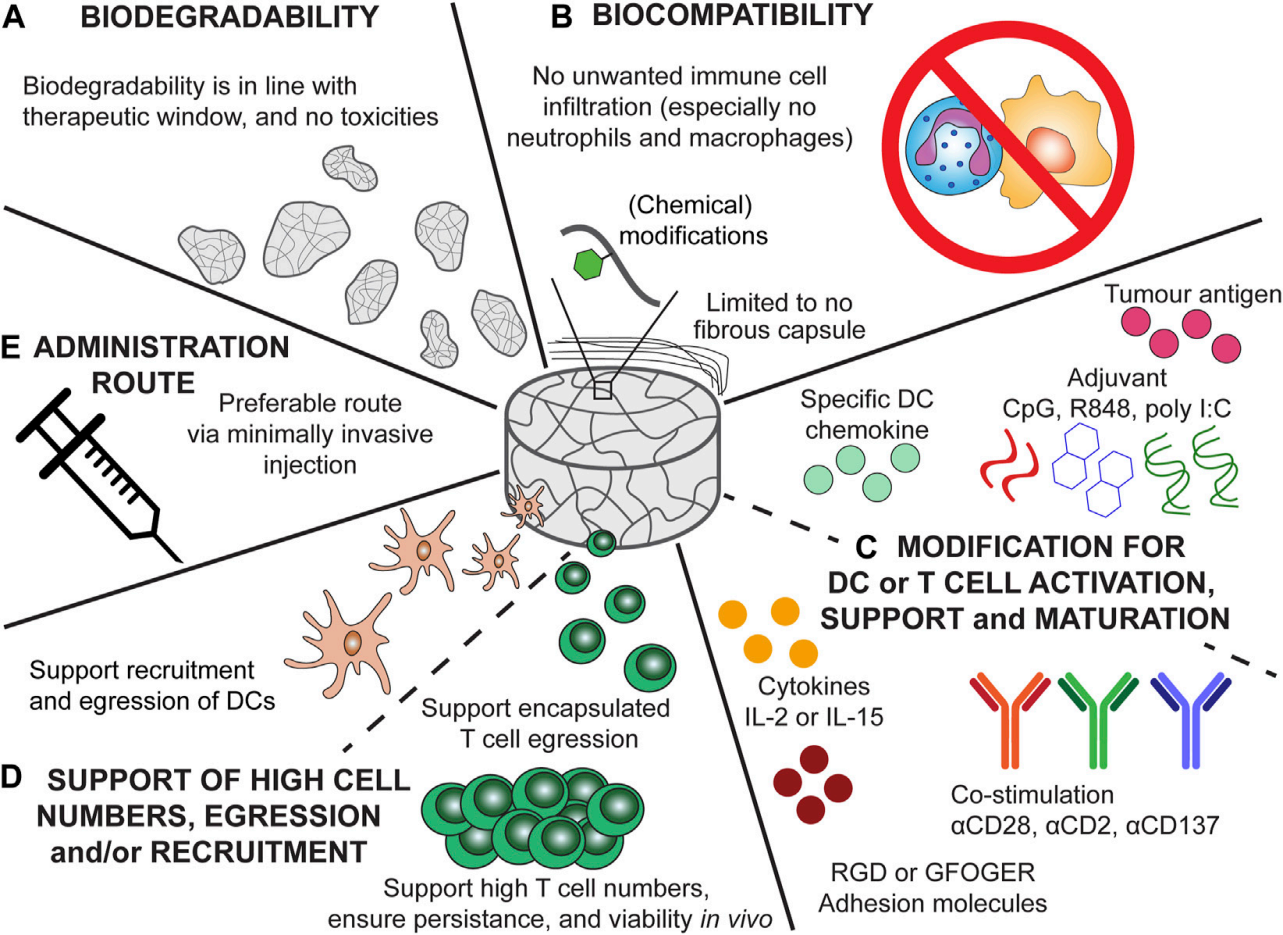
Recommendation for the ideal scaffold design for cancer vaccine scaffolds and scaffolds for ACT of CAR-T cells. **(A)** Biodegradability kinetics should be in accordance with the treatment time, ensuring proper therapeutic outcome and preventing unwanted responses of degradation products. **(B)** Biocompatibility of the scaffolds would ensure only an anti-tumour immune response is elicited and no infiltration of unwanted immune cells (e.g., neutrophils and macrophages) or fibrous encapsulation which can results in scaffold failure. **(C)** Modification of the cancer vaccine scaffold should ensure potent and specific DC recruitment and maturation (by providing tumour antigens and adjuvants). The chemokine incorporated should result in specific DC recruitment. Modification of the scaffolds for ACT should ensure potent T cell activation, viability, and persistence. Especially modification with cytokines (e.g., IL-2 or IL-15), co-stimulatory signals (αCD28, αCD2, or αCD137), and adhesion molecules (GFOGER, RGD) can aid in the persistence and phenotype of T cells. **(D)** The scaffolds for ACT should support high T cell numbers, moreover, the encapsulated T cells should be able to diffuse from the scaffold towards the tumour. The cancer vaccine scaffolds should support the recruitment of DCs and egression of matured DCs. **(E)** Injectable scaffold would be preferable as this would allow for minimally invasive delivery.

Despite encouraging first reports, it is fair to say that both the scaffold-based cancer vaccines and the scaffolds for ACT of CAR-T cells still face challenges regarding the host response. Although initial insights have been obtained as to which scaffold parameters influence the host response, more research is needed to determine exactly which parameters are needed for the optimal scaffold design. Here we hypothesize that the ideal scaffold for anti-tumour immunotherapeutic strategies, such as cancer vaccines and ACT, should encompass the characteristics depicted in Figure 4: a well-tuned biodegradable profile, limited unwanted host response activity, possibilities for modifications to specifically attract and/or activate DCs or T cells, facilitate cell migration, and preferable a non-invasive delivery route. Overall, biomaterials have proven themselves to be useful tools to locally induce an anti-tumour immune response by either actively recruiting DCs or by providing a stimulatory environment post ACT for CAR-T cells.

## Supplementary material

SUPPLEMENTARY FIGURE S1 Chemical structures of the three different triazole alginate modification resulting in reduced FBR. Figure adopted from Vegas et al. (2016)
