# Supplementary material for: Insights in the host response towards biomaterial-based scaffolds for cancer therapy
Source: Front Bioeng Biotechnol. 2023 Jun 5;11:1149943. doi: 10.3389/fbioe.2023.1149943 (PMC10277494; doi:10.3389/fbioe.2023.1149943)
Supplement: Supplementary file 1 [file DataSheet1.docx]

# **Supplemental Information**


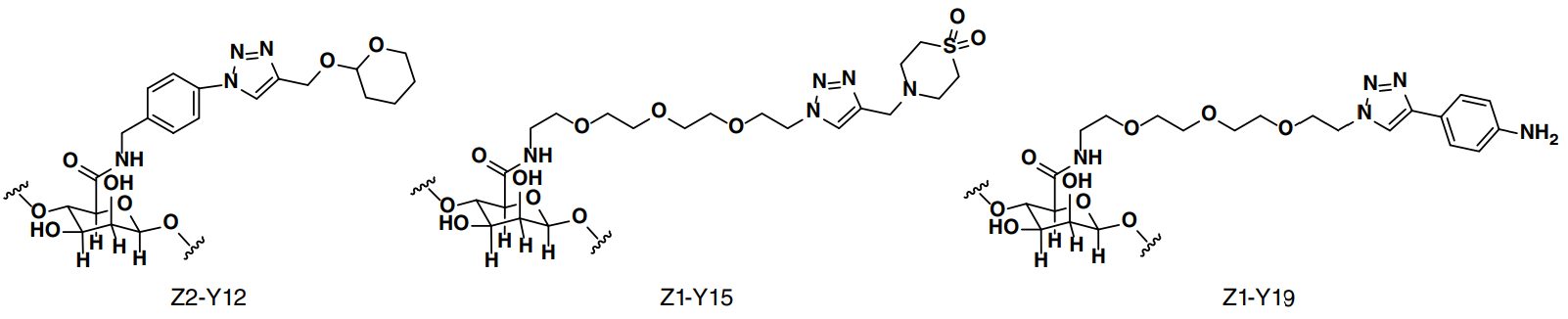


**Supplemental Figure 1. Chemical structures of the three different triazole alginate modification resulting in reduced FBR.** Figure adopted from Vegas et al. (77).
